# The miR-130a-3p/TGF-βRII Axis Participates in Inhibiting the Differentiation of Fibroblasts Induced by TGF-β1

**DOI:** 10.3389/fphar.2021.732540

**Published:** 2021-07-28

**Authors:** Yanhong Liu, Yan Ding, Yapeng Hou, Tong Yu, Hongguang Nie, Yong Cui

**Affiliations:** ^1^ Department of Stem Cells and Regenerative Medicine, College of Basic Medical Science, China Medical University, Shenyang, China; ^2^ Departments of Anesthesiology, The First Hospital of China Medical University, Shenyang, China

**Keywords:** pulmonary fibrosis, mesenchymal stem cells, mouse lung fibroblast, microRNA, TGF-β1

## Abstract

Pulmonary fibrosis (PF) is a chronic progressive interstitial lung disease that has a poor prognosis. Abnormal activation of transforming growth factor-β1 (TGF-β1) plays a crucial role in fibroblast differentiation. Mesenchymal stem cells (MSCs) are currently being considered for the treatment of PF, but the regulatory mechanisms are poorly understood. We co-cultured bone marrow-derived MSCs and mouse lung fibroblasts (MLg) in the presence of TGF-β1, and studied the protein/mRNA expression of fibrosis markers and related signaling pathways. The effects of miR-130a-3p and TGF-β receptor II (TGF-βRII) on the differentiation of MLg induced by TGF-β1 were studied using immunofluorescence assay, Western blot, and quantitative real-time PCR techniques, respectively. Our results showed that MSCs reversed the overexpression of fibrosis markers and TGF-β1/Smad signaling pathway proteins and mRNAs after TGF-β1 treatment and increased the level of miR-130a-3p. TGF-βRII was identified as a target of miR-130a-3p and was evaluated by dual-luciferase reporter assay. The miR-130a-3p/TGF-βRII axis could suppress the differentiation of lung fibroblasts *via* the TGF-β1/Smad signaling pathway, thereby reducing the process of PF.

## Introduction

Pulmonary fibrosis (PF) is a chronic lung disease, and its incidence has been rising in recent years (Wu and Xu, 2020; Li et al., 2021). PF usually affects elderly patients, and radiation and mechanical or chemical stimulation have become the main risk factors associated with PF (Froidure et al., 2020; Jin et al., 2020; Yang et al., 2020). The main pathological features of PF are alveolar epithelial cell damage, fibroblast proliferation and activation, excessive collagen deposition, and a large accumulation of extracellular matrix (Sauler et al., 2019; Zhi et al., 2020). Transforming growth factor-β1 (TGF-β1) regulates proliferation and differentiation in a multitude of cell types, and TGF-β1/Smad signaling pathway is associated with fibrosis (Barrett et al., 2017; Liu et al., 2020; Wnuk and Paw, 2020). TGF-β1 binds to TGF-β receptor II (TGF-βRII) on the cell surface, which recruits TGF-βRI. Activation of the latter can phosphorylate Smad2/3 and associates with Smad4, which translocates from the cytoplasm to the nucleus, and regulate gene transcription (Yoshida et al., 2018; Wang et al., 2021).

Mesenchymal stem cells (MSCs) were first discovered and isolated from bone marrow and have multilineage differentiation and self-renewal capacity (Li and Wu, 2020). MSCs are able to promote cell proliferation, repair tissue damage, and improve the microenvironment of the lung tissue *via* extracellular vesicle release to deliver DNA, mRNA, and microRNAs (miRNAs) (Behnke et al., 2020; Kavianpour and Saleh, 2020; Li et al., 2020). Stem cell-derived miRNAs have recently been found to regulate various cellular processes, such as proliferation, apoptosis, stress responses, differentiation, and angiogenesis. Recent literature has shown the potential roles of miRNAs, a type of small noncoding RNA in PF (Dutta et al., 2019; Boateng and Krauss-Etschmann, 2020; Cho et al., 2020), which can regulate target genes or proteins through inflammatory responses related to mRNA degradation or translation inhibition. Among these miRNAs, miR-130a-3p observed in MSCs has been reported to have antitumor effects and has been used for treating neurological diseases (Chen et al., 2010; Glaesel et al., 2020; Zhong et al., 2020). It has also been found that miR-130a-3p could exert cardioprotective effects through the induction of angiogenesis in ischemic hearts after myocardial infarction (Moghaddam, et al., 2019). In the bleomycin-treated mouse PF model, miR-130a-3p mimics significantly reduced the macrophage profibrogenic gene expression (Su et al., 2015)*.* However, studies focused on miR-130a-3p functions in fibroblasts are seldom published. In this study, we investigated bone marrow MSC (BMSC)-derived miR-130a-3p and co-cultured it with mouse lung fibroblasts (MLg) to explore its expression and regulatory mechanism in PF.

By analysis and prediction using TargetScan and miRanda software, we found that miR-130a-3p and TGF-βRII are potential binding partners, the latter of which is important for the TGF-β1 signaling pathway (Kushibiki et al., 2005). We speculate that BMSC-derived miR-130a-3p may participate in the TGF-β1/Smad signaling pathway to inhibit the differentiation of lung fibroblasts, which provides an effective therapeutic approach for PF prevention and treatment by targeting TGF-βRII.

## Materials and Methods

### BMSC Culture and Co-Cultured With MLg

The male C57 mice (weight 9–10 g) were provided by China Medical University, and all experiments were performed with the research protocol approved by the Ethics Committee of China Medical University (No. CMU2019088). According to the method previously reported, BMSCs were isolated and cultured in DMEM/F12 medium with 10% fetal bovine serum (FBS, Gibco, United States), 10 ng/ml recombinant mouse basic fibroblast growth factor (PeproTech, United States), 1% penicillin/streptomycin, and maintained at 37°C in 5% CO_2_ (Hou et al., 2020). The medium was changed every 2 days. BMSC characterization has been confirmed in our previously published paper (Zhou et al., 2021). At 80% confluence, the medium of BMSCs was changed with DMEM/F12 medium without FBS. BMSC-CM was collected after 24 h and stored at −80°C. MLg was purchased from American Type Culture Collection (ATCC, United States) and cultured in a 6-well plate with DMEM medium adding 10% fetal bovine serum (FBS, Gibco, United States), and 1% penicillin/streptomycin. For the co-culture mode, the BMSCs were prepared in 6-well Transwell inserts, which were transferred to the 6-well plate with MLg seeded at the bottom after 24 h. The BMSCs co-cultured with MLg were treated with DMEM without FBS for another 24 h, and used for the following experiment.

### Cell Transfection

BMSCs or MLg was transfected using Lipofectamine 2000 (GenePharma, CHN) according to the manufacturer’s protocol. In brief, 50–60% confluent BMSCs or MLg grown in medium was incubated with Lipofectamine 2000 reagent and TGF-βRII-siRNA (si-TGF-βRII), miR-130a-3p mimic (Mimic), miR-130a-3p inhibitor (Inhibitor), negative control (NC, the negative control of miR-130a-3p mimic or si-TGF-βRII), and inhibitor NC (the negative control of miR-130b inhibitor) for 72 h, respectively. Labeled CY3-miR-130a-3p was also used and its distribution was observed under a fluorescence microscope.

### Western Blot Assays

MLg was washed three times with PBS and lysed using a cell lysis reagent. Proteins were analyzed by SDS-PAGE (10% polyacrylamide gels) and transferred to PVDF membranes (Invitrogen, United States). Membranes were blocked with 5% BSA for 1 h and incubated with primary antibodies, which were against α-SMA (Merck, GER; 1:1,000 dilution), FN (Abcam, United States; 1:1,000 dilution), TGF-βRII (Elabscience, CHN; 1:1,000 dilution), SMAD2/3 (Elabscience, CHN; 1:1,000 dilution), p-SMAD2/3 (Elabscience, CHN; 1:1,000 dilution), and β-actin (Santa Cruz, United States; 1:1,000 dilution). The membranes were then washed three times with TBST and incubated with goat anti-rabbit or anti-mouse secondary antibody (ZSGB-BIO, CHN; 1:5,000 dilution) at room temperature for 1 h. The bands of protein were obtained from the same membrane, which were then visualized using an ECL kit on a Tanon-5200 chemiluminescence detection system (Tanon, CHN) and quantified using ImageJ program.

### Quantitative Real-Time PCR Analysis

According to the manufacturer’s instructions, total RNA was isolated using TRIzol reagent (Invitrogen, United States) and the extracted RNA samples were quantified by NanoDrop 2000C spectrophotometer (Thermo, United States). Total RNA and miRNAs were reverse transcribed into cDNA using synthesis Kit (TaKaRa, Japan). Quantitative real-time PCR (qRT-PCR) reactions were then performed with SYBR Premix Ex Taq II (TaKaRa, Japan) in the ABI 7500 System. Results were calculated using the GAPDH (for mRNAs) or U6 gene (for miRNAs) (Santa Cruz Biotechnology, United States) as internal controls based on the 2^−ΔΔCT^ method. All the primers are shown in Table 1.

**TABLE 1:** Primer sequences of qRT-PCR.

Gene	Forward primer sequence	Reverse primer sequence
*α-SMA*	5′-CTA TGA AGG CTA TGC CCT GCC-3′	5′-GCT TCT CCT TGA TGT CTC GCA C-3′
*FN*	5′-TGT TAT GGA GGA AGC CGA GGT T-3′	5′-CGA TGC AGG TAC AGT CCC AGA-3′
*Col III*	5′-TGG AAA CTG GGG AAA CAT GC-3′	5′-GGA TTG CCG TAG CTA AAC TGA-3′
*TGF-βR I*	5′-AAA CTT GCT CTG TCC ACG G-3′	5′-AAT GGC TGG CTT TCC TTG-3′
*TGF-βR II*	5′-GTA ATA GGA ATG CCC ATC CAC-3′	5′-GAT TTC TGG TTG TCA CAG GTG-3′
*SMAD2*	5′-AGA ATA TCG GAG GCA GAC AG-3′	5′-GTT ACA GCG AGT CTT TGA TGG-3′
*GAPDH*	5′-AGA AGG CTG GGG CTC ATT TG-3′	5′-AGG GGC CAT CCA CAG TCT TC-3′
*U6*	5′-GGA ACG ATA CAG AGA AGA TTA GC-3′	5′-TGG AAC GCT TCA CGA ATT TGC G-3′
miR-130a-3p	5′-CAG UGC AAU GUU AAA AGG GCA U-3′	5′-GCC CUU UUA ACA UUG CAC UGU U-3′

### Cell Viability Assay

The effects of miR-130a-3p and si-TGF-βRII on MLg viability were measured by CCK8 assay (Dojindo, CHN) according to the manufacturer’s instructions. Briefly, MLg were cultured in a 96-well plate along with NC, miR-130a-3p, or si-TGF-βRII, respectively. After 24 h, TGF-β1 (5 μl) was added to the corresponding well. 48 h after incubation, 10% CCK8 solution was added to each well. After the cells were incubated for additional 2 h, the absorbance was measured at 450 nm wavelength with a microplate reader (Tecan, CH).

### Immunofluorescence Staining for α-SMA

MLg was incubated in 6-well plates, and fixed for 30 min with 4% of paraformaldehyde (Sigma, GER). After washing, 0.1% Triton X-100 was used for permeabilization. The cells were then visualized using anti-α-SMA primary antibody (CBL171, Mreck, GER; 1:200 dilution) at 4°C overnight and a labeled secondary antibody for 90 min. The nuclei were stained for DAPI for 15 min in the dark. Finally, the cells were examined using a fluorescence microscope.

### Dual Luciferase Reporter Gene Assay

The wild-type (WT) TGF-βRII 3ʹ-untranslated regions (3ʹ-UTR) and mutant-type (MUT) TGF-βRII (3ʹ-UTR) sequences of TGF-βRII were synthesized by GenePharma (CHN). The miR-130a-3p mimic or miR-130a-3p negative control was cotransfected into H441 cells using Lipofectamine 2000. After 48 h, luciferase activities were detected by the Dual Luciferase Reporter Assay Kit (Vazyme, CHN).

### Statistical Analysis

All data were processed by Origin 8.0 and presented as the mean ± SE. Normality and homoscedasticity test were done by Levene and Shapiro-Wilk test before applying parametric tests. The differences between two groups was determined by Student’s two-tailed *t*-test. When the data did not pass the normality or homoscedasticity test, non-parametric *t*-test (Mann-Whitney *U*-test) was applied. *p* < 0.05 was regarded as statistically significant.

## Results

### BMSCs Rescues TGF-β1-Induced Myofibroblast Differentiation *In Vitro*

**FIGURE 1:**
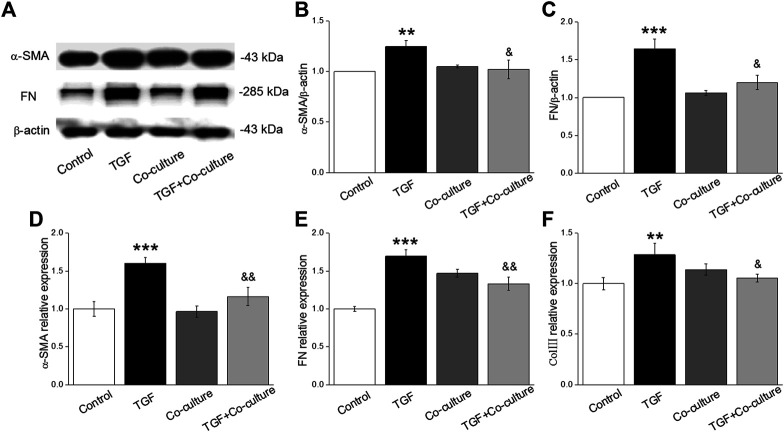
TGF-β1 treatment enhances the level of fibrotic-related protein/mRNAs and BMSCs reverse the above expression in MLg. **(A)** Representative bands of α-SMA and FN protein expression by Western blot in MLg treated with TGF-β1 for 24 h and/or co-cultured with BMSCs for 24 h **(B,C)** Statistical analyses were showed with relative gray values of the protein bands (α-SMA/β-actin and FN/β-actin). **(D-F)** Representative statistical analysis of α-SMA, FN and Col III mRNA by qRT-PCR (α-SMA/GAPDH, FN/GAPDH and Col III/GAPDH). Data were presented as mean ± SE, ^**^ *p* < 0.01, ^***^ *p* < 0.001, compared with Control group. ^&^ *p* < 0.05, ^&&^ *p* < 0.01, compared with TGF group. *n* = 3–6.

TGF-β1 is involved in the development of PF. Some studies have shown that the inhibition of proliferation and differentiation of fibroblasts are related to the activation of TGF-β1, and the signaling pathways mediated by it play a vital role in lung diseases (Wolters et al., 2014; Aschner and Downey, 2016). The PF model was constructed by treating MLg with 10 ng/mL TGF-β1 for 24 h, which in some case was also added just after MLg was co-cultured with BMSCs. The Western blot assay revealed that the levels of fibrosis markers (α-SMA and FN) in MLg increased after TGF-β1 treatment, whereas the expression levels reduced when co-cultured with BMSCs (Figures 1A–C), indicating a successful PF *in vitro* model with TGF-β1-induction. Subsequently, we assessed the expression levels of the above indicators with qRT-PCR, and the result was consistent with that of the Western blot (Figures 1D–F). These data suggest that BMSCs significantly rescued TGF-β1-induced myofibroblast differentiation.

**FIGURE 2:**
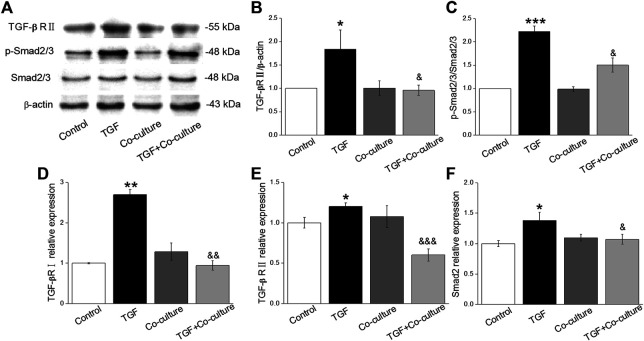
BMSCs decrease the level of TGF-β1/Smad signaling pathway proteins in MLg treated with TGF-β1. **(A)** Representative bands for TGF-βRII, Smad2/3, and p-Smad2/3 protein levels. **(B,C)** Statistical analyses were obtained from relative gray values of the protein bands (TGF-βRII/β-actin and p-Smad2/3/Smad2/3). **(D-F)** Representative statistical analysis of TGF-βRI, TGF-βRII and Smad2 mRNA by qRT-PCR (TGF-βRI/GAPDH, TGF-βRII/GAPDH and Smad2/GAPDH). Data were presented as mean ± SE, ^*^ *p* < 0.05, ^**^ *p* < 0.01, ^***^ *p* < 0.001, compared with Control group. ^&^ *p* < 0.05, ^&&^ *p* < 0.01, ^&&&^ *p* < 0.001, compared with TGF group. *n* = 3–4.

Aberrant transcription of signaling pathways might trigger PF, among which TGF-β1 plays a central role (Saito et al., 2018a; Froidure et al., 2020). As expected, TGF-β1/Smad signaling pathway proteins (TGF-βRII, SMAD2/3 and p-SMAD2/3) increased with TGF-β1 treatment, which was reversed after BMSC administration (Figures 2A–C), and verified at the mRNA level (Figures 2D–F). This observation indicates that the TGF-β1/Smad signaling pathway may participate in the beneficial effect of BMSCs in PF.

### BMSCs Enhance the Expression Level of miR-130a-3p After TGF-β1 Induction

**FIGURE 3:**
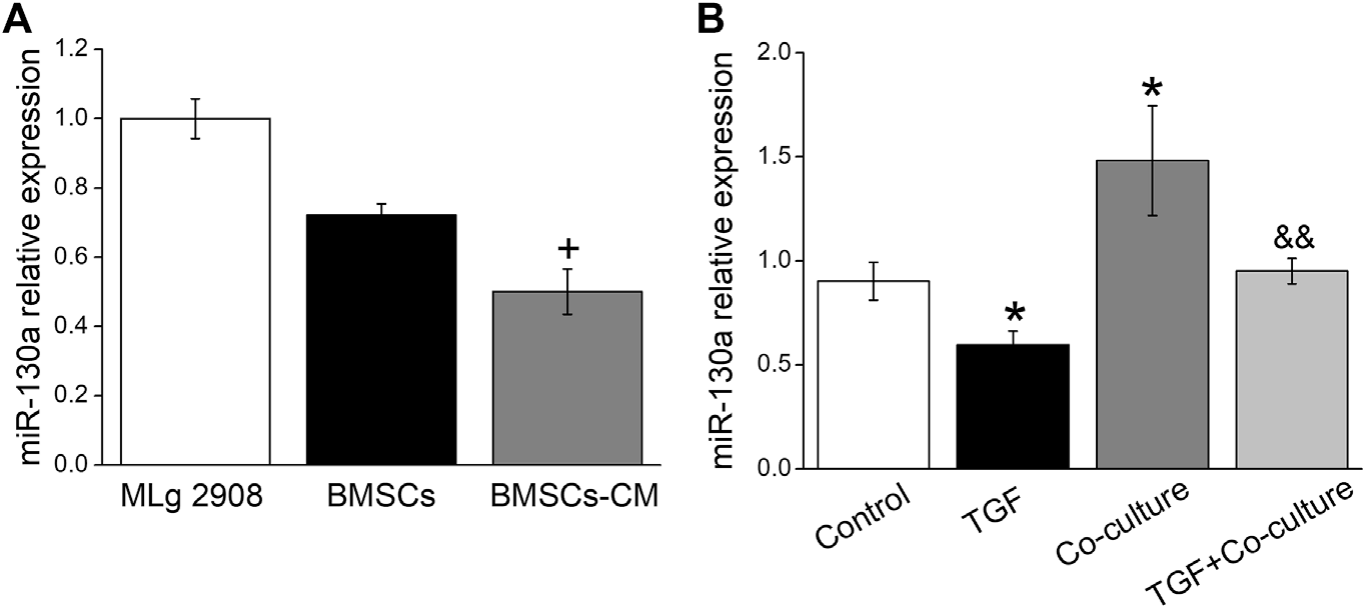
The expression of miR-130a-3p *in vitro*. **(A)** The expression of miR-130a-3p in MLg, BMSCs and BMSCs-CM was detected by qRT-PCR (miR-130a-3p/U6). **(B)** The expression of miR-130a-3p in MLg treated with TGF-β1 for 24 h and/or co-cultured with BMSCs for 24 h ^+^ *p* < 0.05, compared with BMSCs group. ^*^ *p* < 0.05, compared with Control group. ^&&^ *p* < 0.01, compared with TGF group. *n* = 3.

It is well known that miRNAs can help MSCs communicate with their surrounding environment (Asgarpour et al., 2020). We hypothesized that the role of BMSCs in TGF-β1-induced fibroblasts may be mediated *via* miRNAs. Based on relevant literature reports and website prediction, we screened out miR-130a-3p that may be beneficial to PF, which was present both in MSCs and MSCs-derived microparticles (Chen et al., 2010; Ferguson, et al., 2018). BMSC-CM is a medium that contains the secretions in BMSCs, such as exosome, miRNAs, etc (Marlina, et al., 2021). We postulated that BMSCs may participate in the protective effects on PF by secreting miR-130a-3p to BMSC-CM and then exert its effects on the receptor cell MLg, and to verify this possibility, we compared the content of miR-130a-3p originating from MLg, BMSC, and BMSC-CM, which showed higher expression levels in BMSC than in BMSC-CM (Figure 3A). Moreover, miR-130a-3p significantly decreased after TGF-β1 induction compared with the control group, which was reversed after BMSC administration, suggesting that miR-130a-3p might be related to BMSC treatment of PF (Figure 3B).

### MiR-130a-3p Inhibits Fibroblast Differentiation

**FIGURE 4:**
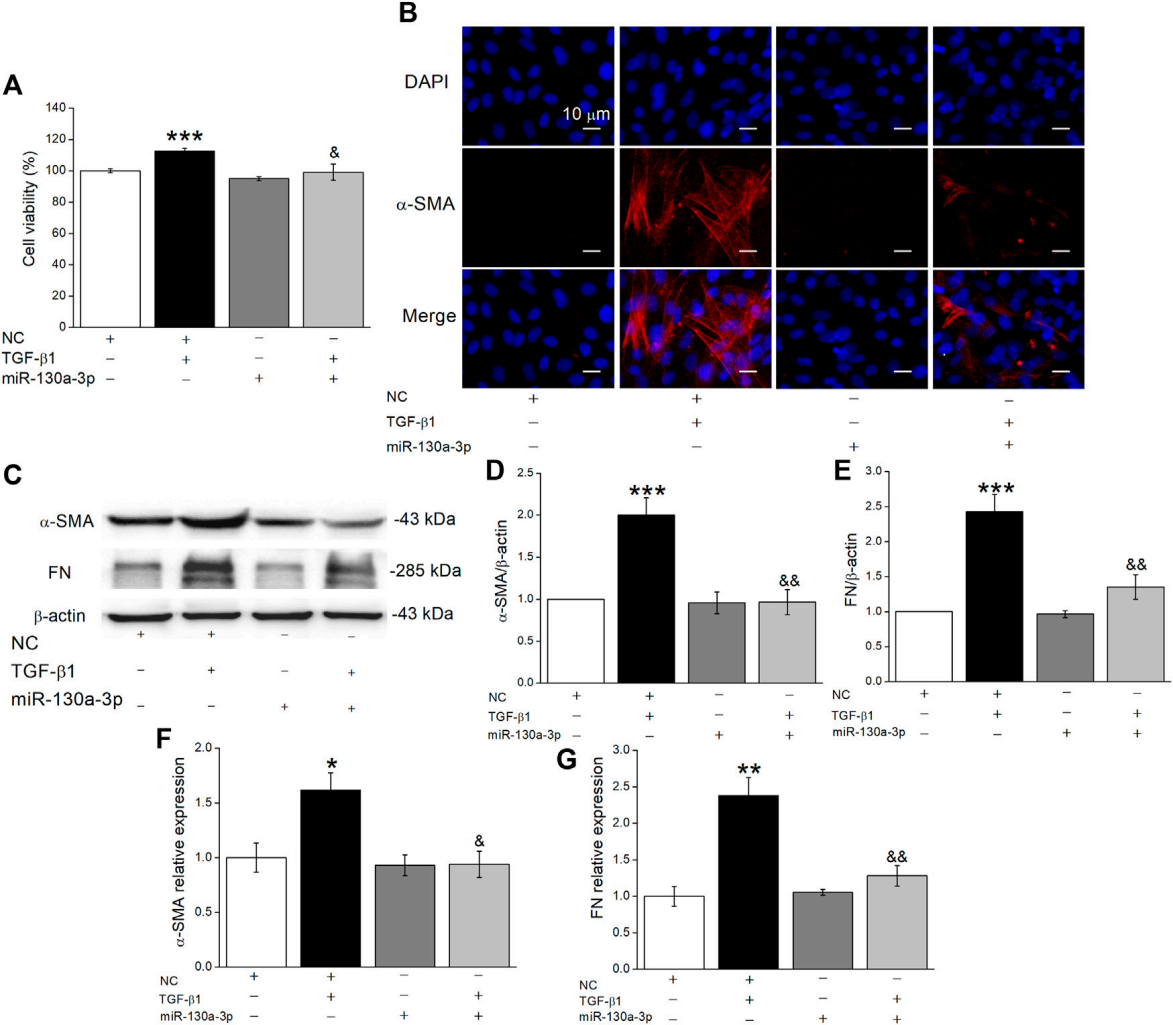
The effect of miR-130a-3p on the differentiation in TGF-β1-induced MLg. **(A)** The relative cell viability was measured in CCK8 assay, and the data for NC group was set to 100%. **(B)** The effect of miR-130a-3p on fibroblast-myofibroblast transition was measured with immunofluorescence. Scale bar = 10 μm. **(C)** Western blot bands of α-SMA and FN protein expression in MLg after transfection of miR-130a-3p. **(D,E)** Statistical analyses were obtained from relative gray values of the protein bands (α-SMA/β-actin and FN/β-actin). **(F,G)** The result of qRT-PCR assay shows the α-SMA and FN mRNA level in MLg (α-SMA/GAPDH, and FN/GAPDH). Data were presented as mean ± SE, ^*^ *p* < 0.05, ^**^ *p* < 0.01, ^***^ *p* < 0.001, compared with NC group. ^&^ *p* < 0.05, ^&&^ *p* < 0.01, compared with NC + TGF group. *n* = 3–5.

Increasing evidence indicates that both the proliferation and differentiation of fibroblasts contribute to the formation of fibrosis, and TGF-β1 stimulates fibrotic-related α-SMA expression, collagen synthesis, and extracellular matrix deposition (Li et al., 2016; Huang et al., 2017). To confirm the effect of miR-130a-3p, we transfected MLg with the miR-130a-3p mimic and then added TGF-β1 after 48 h. Above all, the CCK8 assay results showed that TGF-β1 significantly promoted the viability in MLg, which was inhibited by miR-130a-3p (Figure 4A). Meanwhile, the immunofluorescence results showed that overexpression of miR-130a-3p inhibited the differentiation of MLg, which was enhanced after TGF-β1 stimulation (Figure 4B). Consistently, TGF-β1 upregulated the expression of fibrotic-related proteins and mRNAs in MLg, which was reversed by the miR-130a-3p mimic (Figures 4C–G). Taken together, these results indicate that miR-130a-3p is involved in the regulation of fibroblast differentiation.

### TGF-βRII is a Target Gene of miR-130a-3p

**FIGURE 5:**
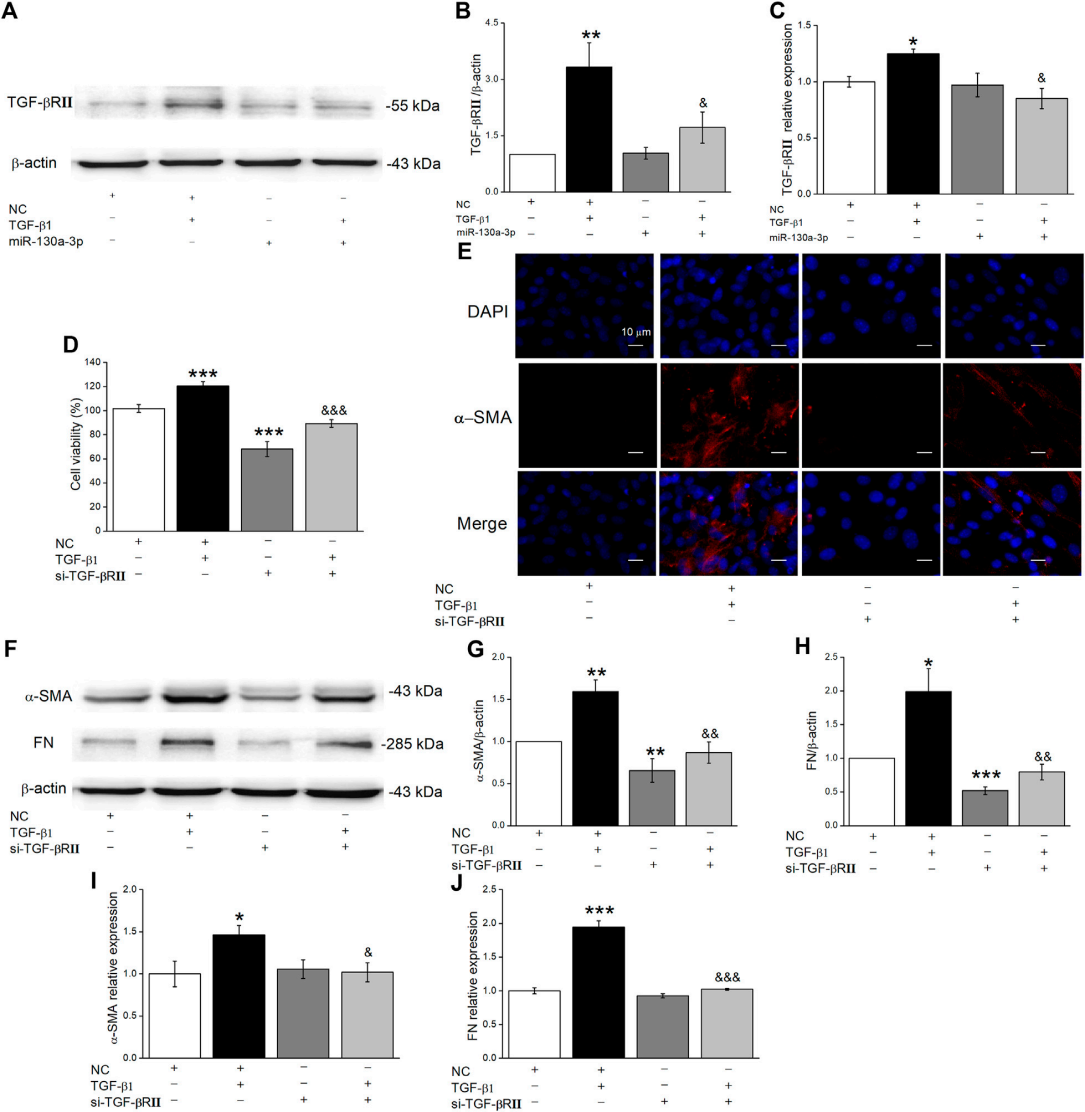
MiR-130a-3p decreases protein level TGF-βRII in MLg and silencing TGF-βRII inhibits TGF-β1-induced MLg differentiation. **(A)** Representative Western blot bands of TGF-βRII protein expression in MLg transfected with miR-130a-3p mimic and treated with TGF-β1 for 24 h. **(B)** Level of TGF-βRII protein was obtained from Western blot analysis. **(C)** Level of TGF-βRII mRNA was analyzed. **(D)** The effect of TGF-βRII knockdown on the inhibition of MLg proliferation. **(E)** Immunofluorescence staining was demonstrated the effect of TGF-βRII silence on MLg differentiation. Scale bar = 10 μm. **(F)** Western blot bands of α-SMA and FN protein expression in MLg after transfection of si-TGF-βRII. **(G,H)** Western blot statistical analyses for α-SMA and FN protein (α-SMA/β-actin and FN/β-actin). **(I,J)** qRT-PCR statistical analyses for α-SMA and FN mRNA (α-SMA/GAPDH and FN/GAPDH). Data were presented as mean ± SE, ^*^ *p* < 0.05, ^**^ *p* < 0.01, ^***^ *p* < 0.001, compared with NC group. ^&^ *p* < 0.05, ^&&^ *p* < 0.01, ^&&&^ *p* < 0.001, compared with NC + TGF group. *n* = 3–4.

Gene targets of miR-130a-3p were identified using miRNA target prediction databases, and we found that TGF-βRII was a possible target gene of miR-130a-3p. Previous studies showed that the TGF-β1 signaling pathway controls cell proliferation, differentiation, organ development, etc., by combing with TGF-βRII (Shi et al., 2020). First, we examined whether miR-130a-3p downregulated TGF-βRII expression by Western blot and qRT-PCR (Figures 5A–C). Moreover, to explore whether miR-130a-3p suppresses the differentiation of MLg by deregulating TGF-βRII, we researched the influence of silencing TGF-βRII on the differentiation of MLg. Using the CCK8 assay, we found that si-TGF-βRII could inhibit the viability of MLg induced by TGF-β1, indicating that TGF-βRII plays an indispensable role in the TGF-β1 signaling pathway (Figure 5D). Simultaneously, si-TGF-βRII was further verified to inhibit differentiation in MLg according to an immunofluorescence assay (Figure 5E). Finally, as shown in Figures 5F–J, fibrosis markers and relative signaling pathways were examined at the protein and mRNA levels, all of which supported that miR-130a-3p could regulate cell differentiation by targeting TGF-βRII.

**FIGURE 6:**
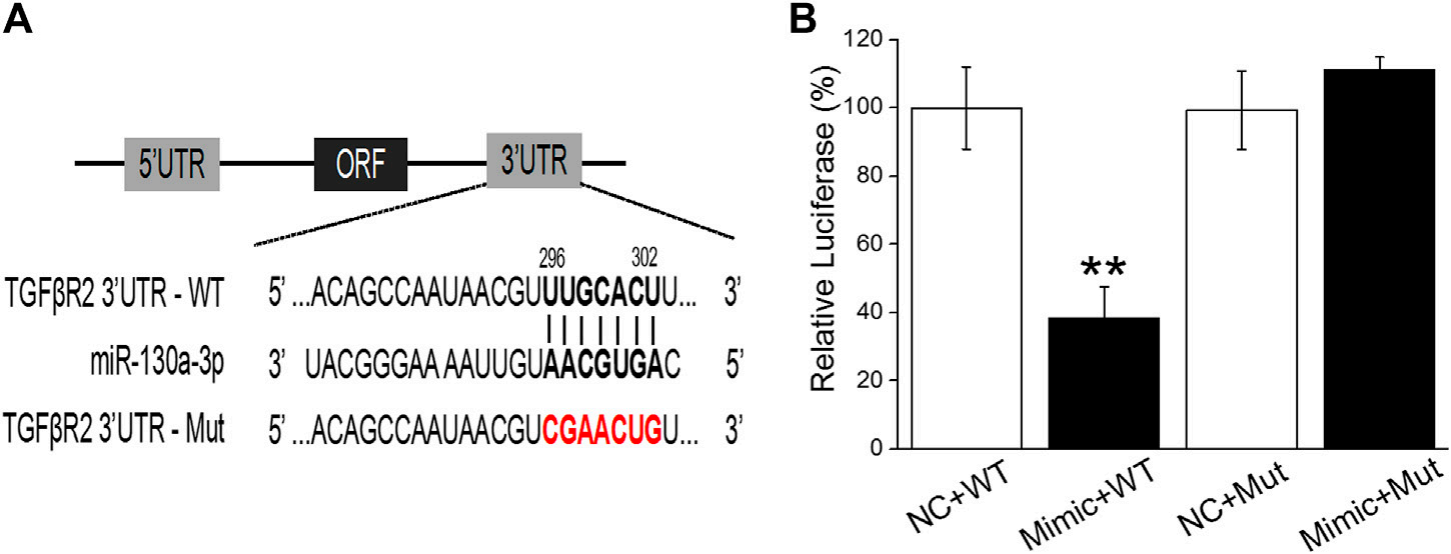
TGF-βRII is a one of target genes of miR-130a-3p. **(A)** The possible binding sites of miR-130a-3p with TGF-βRII-3′UTR. **(B)** Dual-luciferase reporter assays of miR-130a-3p and TGF-βRII. Data were presented as mean ± SE, **p < 0.01, compared with NC + WT group. *n* = 3.

To further gain evidence regarding miR-130a-3p directly targeted TGF-βRII, we cotransfected NC and the miR-130a-3p mimic (Mimic) into H441 cells with a luciferase reporter system containing the wild-type (WT) or mutated (Mut) 3′-UTR of TGF-βRII (Figures 6A,B). The luciferase activity was decreased in H441 cells cotransfected with miR-130a-3p mimic and TGF-βRII-WT (Mimic + WT), compared with H441 cells cotransfected with NC and TGF-βRII-WT (NC + WT). However, the expression of TGF-βRII-Mut (Mimic + Mut) was not suppressed. These data suggest that TGF-βRII is a direct target gene of miR-130a-3p, which could regulate the differentiation of lung fibroblasts by directly interacting with the 3′-UTR of TGF-βRII.

### MiR-130a-3p is Transferred to MLg When Co-Cultured With BMSCs

**FIGURE 7:**
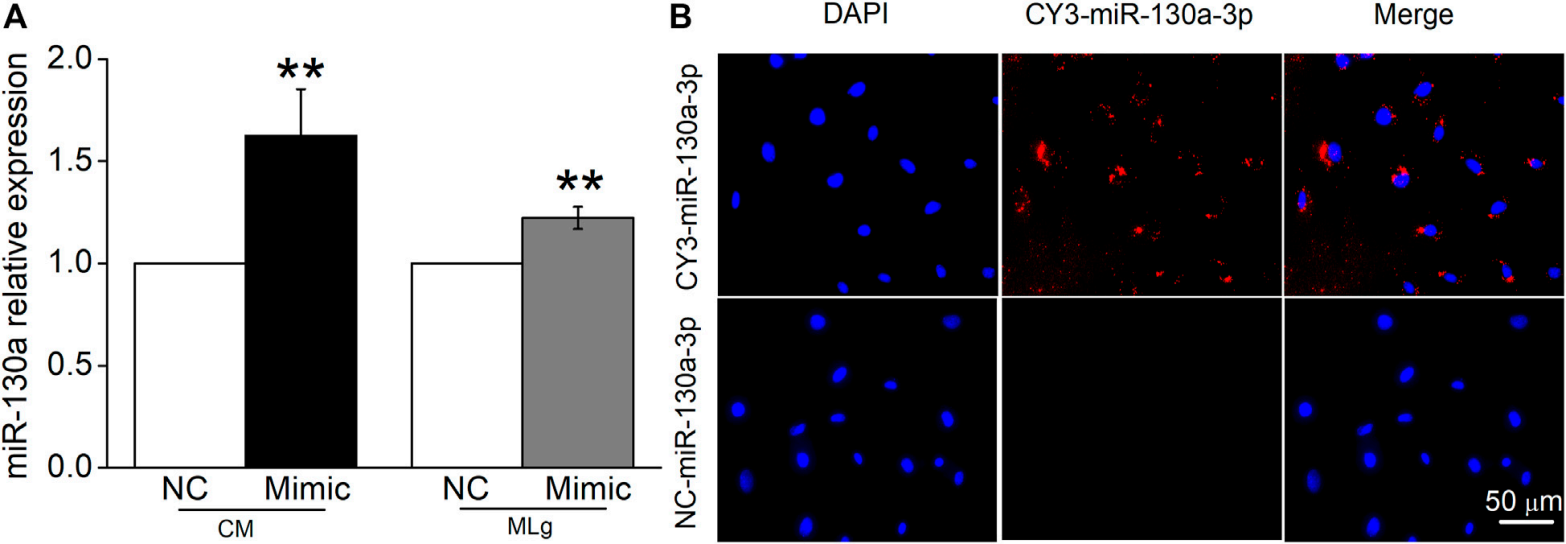
MiR-130a-3p can be transferred to MLg co-cultured with BMSC. **(A)** MiR-130a-3p mimic (Mimic) or negative control (NC) was transfected in BMSCs, and the transfection efficiency was shown in BMSCs-CM (CM) and co-cultured MLg (MLg), respectively. **(B)** BMSCs were transfected with CY3-miR-130a-3p, and co-cultured with MLg for 24 h. The Nuclei were counterstained with DAPI (blue), and a representative CY3-miR-130a-3p stained image was shown in the MLg (red). Scale bar, 50 μm. Data were expressed as mean ± SE, ^**^ *p* < 0.01, compared with NC group. *n* = 3–6.

There is increasing evidence that miRNAs secreted by MSCs play their roles by entering into the receptor cells. We transfected miR-130a-3p mimic or NC into BMSCs, then collected BMSCs-CM after 24 h, and verified the expression of miR-130a-3p in BMSCs-CM by qRT-PCR. Meanwhile, the transfected BMSCs were co-cultured with MLg, and the expression of miR-130a-3p was also detected in MLg (Figure 7A). In order to provide direct evidence that miR-130a-3p in BMSCs can be transferred to MLg, we transfected fluorescently labeled CY3-miR-130a-3p into BMSCs and observed its distribution in the co-cultured MLg under a fluorescence microscope. The evidence of CY3-miR-130a-3p appearance in MLg co-cultured with BMSCs, provided us a proof that at least miR-130a-3p from BMSCs could be transferred to the receptor cell MLg, and then exerted its effects on PF (Figure 7B).

### MiR-130a-3p from BMSCs Participates in the Regulation of Fibrosis Through the TGF-β1/Smad Signaling Pathway

**FIGURE 8:**
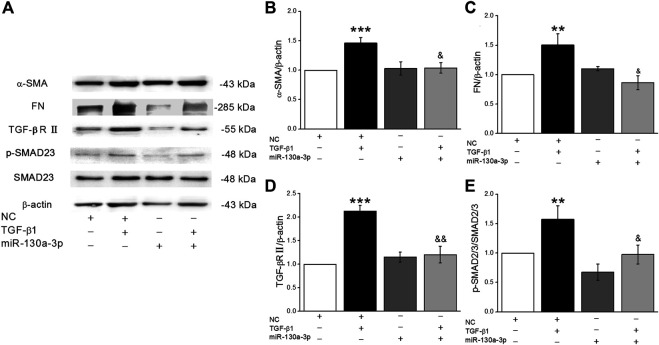
MiR-130a-3p in BMSCs regulates fibrogenesis of MLg induced by TGF-β1 by inhibiting the function of TGF-βRII. **(A)** Representative Western blot bands of α-SMA, FN, TGF-βRII, Smad2/3 and p-Smad2/3 protein expression in BMSCs transfected with NC or miR-130a-3p mimic, followed by co-cultured with MLg. **(B–E)** The statistical analysis of the above-mentioned proteins was obtained from the relative gray values of the protein bands (α-SMA/β-actin, FN/β-actin, TGF-βRII/β-actin, p-Smad2/3/Smad2/3). Data were presented as mean ± SE, ^**^ *p* < 0.01, ^***^ *p* < 0.001, compared with NC group. ^&^ *p* < 0.05, ^&&^ *p* < 0.01, compared with NC + TGF group. *n* = 3–4.

To investigate whether miR-130a-3p from BMSCs could ameliorate the expression of fibrosis markers, we transfected the miR-130a-3p mimic into BMSCs, which were then co-cultured with MLg. As shown in Figure 8 A-E, MLg treated with TGF-β1 increased expression of α-SMA, FN, TGF-βRII, and p-SMAD2/3, all of which were reversed after administration of miR-130a-3p. The above data suggested that BMSCs may participate in the development of PF through miR-130a-3p, which could regulate the TGF-β1/Smad signaling pathway by targeting TGF-βRII.

## Discussion

PF is a progressive interstitial pneumonia characterized by excessive proliferation of fibroblasts and deposition of the extracellular matrix. Currently, the overall prognosis is poor, and effective therapeutic strategies are scarce (Noble et al., 2012; Wang et al., 2016; Chanda et al., 2019). Fibroblasts are regarded as the key effector cells of PF, involved in the key process of pathogenesis (Wuyts et al., 2013). Among them, TGF-β1/Smad signaling participates in the lung epithelium mesenchyme transition, and contributes to the process of PF (Saito et al., 2018b; Liu et al., 2018). In recent years, there has been much enthusiasm regarding MSC therapy for the treatment of lung diseases (Srour and Thébaud, 2015), and there are reports that the beneficial effects of MSCs may be mediated mainly through paracrine action (Willis et al., 2018). In this study, we first demonstrated the impact of BMSCs on MLg with Western Blot and qRT-PCR, and the results showed that compared with the control group, the expression of fibrosis marker and TGF-β1/Smad signaling pathway proteins and mRNAs increased in MLg after using TGF-β1 alone, whereas the effect was opposite after the coadministration of BMSCs. These results confirmed that BMSCs may contribute to alleviating fibrosis formation during PF, the mechanism of which is related to the regulation of the TGF-β1/Smad signaling pathway.

MiRNAs function by binding to the 3′-untranslated regions of mRNA to regulate posttranscriptional processes, thereby controlling various cellular processes (Su et al., 2015; Kang, 2017). We speculate that the above results may also work through miRNAs and evaluate the role of miR-130a-3p, which has been reported to reduce cell senescence and regulate the epithelial-mesenchymal transition (Jiang et al., 2020; Yin et al., 2020). However, it has been relatively seldom studied in TGF-β1-induced lung fibroblasts or PF. First, we confirmed the expression of miR-130a-3p in BMSC-CM and BMSCs by qRT-PCR. In addition, miR-130a-3p was downregulated by treatment with TGF-β1 in MLg, and when co-cultured with BMSCs, the expression level was significantly increased, suggesting that miR-130a-3p derived from BMSCs could influence TGF-β1-induced MLg. Next, overexpression of miR-130a-3p inhibited the cell differentiation phenotype. Future studies are warranted to explore the *in vivo* effects of miR-130a-3p in a bleomycin-induced PF animal model and its translational value as a diagnostic marker and therapeutic target for PF.

**FIGURE 9:**
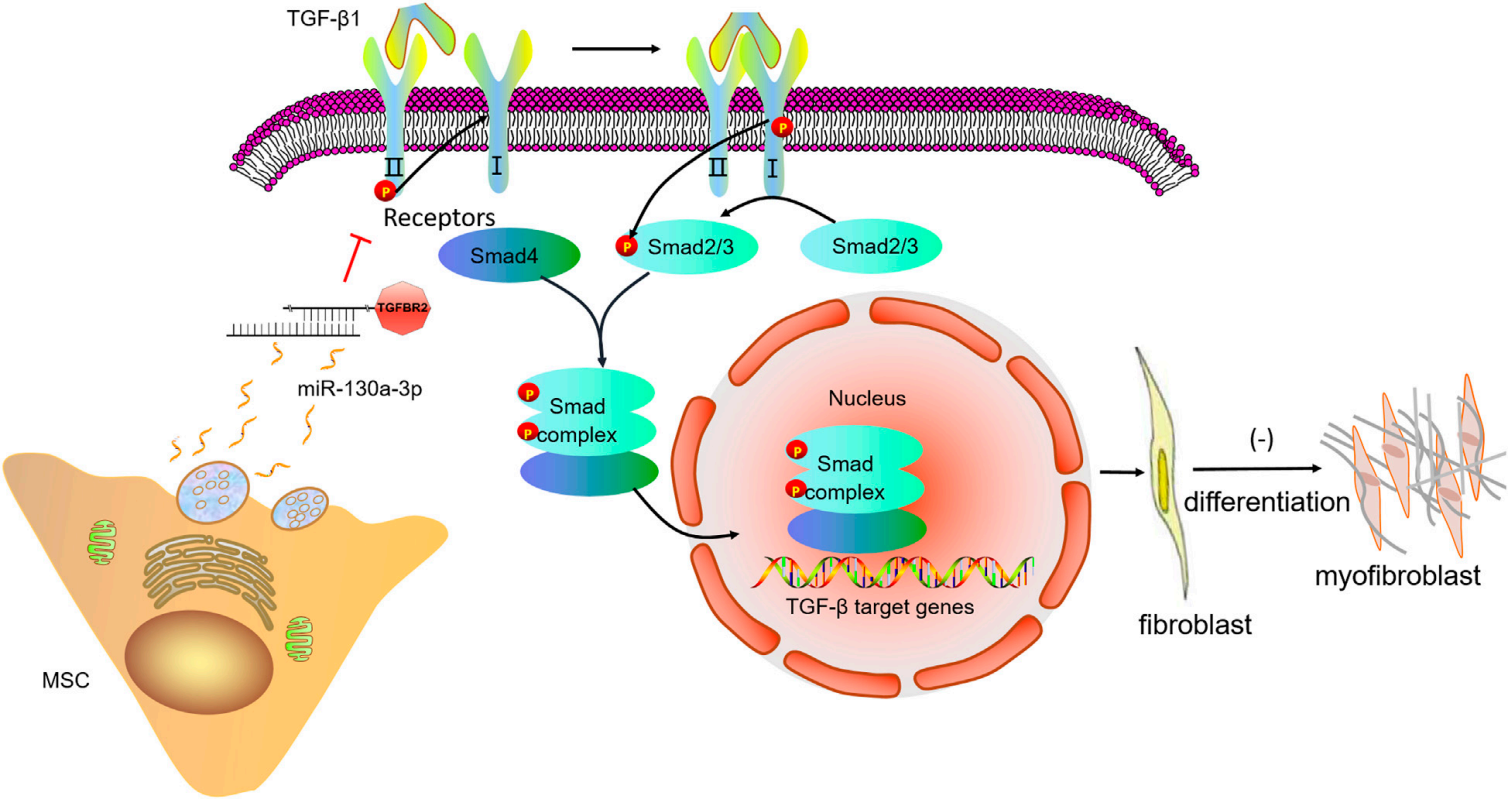
A schematic diagram highlighting the regulation of the cell differentiation by miR-130a-3p in MSCs. The miR-130a-3p secreted by MSCs directly binds to the 3′UTR of TGF-βRII, thereby down-regulating TGF-βRII and inhibiting the recruitment and activation of TGF-βRI, subsequently, phosphorylation of Smad2/3 is inhibited, and unable to form a complex with Smad4, which translocates to the nucleus to regulate gene transcription. MSC, mesenchymal stem cells; TGF-β1, transforming growth factor-β1; TGFBR2, transforming growth factor-β receptor II.

Of note, TGF-βRII has been predicted by relevant websites and verified as a potential target of miR-130a-3p using dual-luciferase reporter system in our study. Moreover, TGF-βRII was downregulated after miR-130a-3p overexpression, consistent with the direct targeting role of TGF-βRII for miR-130a-3p. Previous studies have shown that TGF-βRII-deficient mice could effectively resist PF caused by bleomycin (Li et al., 2011), supporting that as a receptor necessary for TGF-β1 signaling, TGF-βRII is essential for the development of PF (Li et al., 2011; Zhang et al., 2017). After the induction model was constructed by transfection of TGF-βRII siRNA, we found that cell differentiation were inhibited, and the expression of fibrosis marker proteins and mRNAs was reduced, indicating that silencing TGF-βRII had similar effects on the overexpression of miR-130a-3p in the process of affecting fibroblasts. We also verified that BMSCs transmit miR-130a-3p into TGF-β1-induced MLg to target the TGF-βRII axis, thereby inactivating the TGF-β1/Smad signaling pathway (Figure 9), which may provide a future direction for the treatment of PF.

## Conclusion

MiR-130a-3p in BMSCs inhibits the lung fibroblast differentiation through blocking the activation of TGF-β1/Smad signaling pathway.

## Data Availability

The raw data supporting the conclusion of this article will be made available by the authors, without undue reservation.

## Abbreviations

miRNAs, microRNAs; MLg, mouse lung fibroblast; MSCs, mesenchymal stem cells; PF, pulmonary fibrosis; qRT-PCR, quantitative real-time PCR; TGF-β1, transforming growth factor-β1; TGF-βRII, TGF-β receptor II.
